# Effects of Strand Lay Direction and Crossing Angle on Tribological Behavior of Winding Hoist Rope

**DOI:** 10.3390/ma10060630

**Published:** 2017-06-09

**Authors:** Xiang-dong Chang, Yu-xing Peng, Zhen-cai Zhu, Xian-sheng Gong, Zhang-fa Yu, Zhen-tao Mi, Chun-ming Xu

**Affiliations:** 1 School of Mechanical and Electrical Engineering, China University of Mining and Technology, Xuzhou 221116, China; Changxd@cumt.edu.cn (X.-d.C.); zhuzhencai@cumt.edu.cn (Z.-c.Z.); mizhentao@cumt.edu.cn (Z.-t.M.); ts16050066a3@cumt.edu.cn (C.-m.X.); 2 Jiangsu Key Laboratory of Mine Mechanical and Electrical Equipment, China University of Mining & Technology, Xuzhou 221116, China; 3 College of Mechanical Engineering, Chongqing University, Chongqing 400044, China; cqxsgong@cqu.edu.cn; 4 CITIC Heavy Industries Co. Ltd., Luoyang 471039, China; citichic006@126.com; 5 Luoyang Mining Machinery Engineering Design Institute, Luoyang 471039, China

**Keywords:** wire rope, strand lay direction, crossing angle, friction, wear, 81.05.Bx, 81.70.Bt, 81.70.Pg

## Abstract

Friction and wear behavior exists between hoisting ropes that are wound around the drums of a multi-layer winding hoist. It decreases the service life of ropes and threatens mine safety. In this research, a series of experiments were conducted using a self-made test rig to study the effects of the strand lay direction and crossing angle on the winding rope’s tribological behavior. Results show that the friction coefficient in the steady-state period shows a decreasing tendency with an increase of the crossing angle in both cross directions, but the variation range is different under different cross directions. Using thermal imaging, the high temperature regions always distribute along the strand lay direction in the gap between adjacent strands, as the cross direction is the same with the strand lay direction (right cross contact). Additionally, the temperature rise in the steady-state increases with the increase of the crossing angle in both cross directions. The differences of the wear scar morphology are obvious under different cross directions, especially for the large crossing angle tests. In the case of right cross, the variation range of wear mass loss is larger than that in left cross. The damage that forms on the wear surface is mainly ploughing, pits, plastic deformation, and fatigue fracture. The major wear mechanisms are adhesive wear, and abrasive and fatigue wear.

## 1. Introduction

With the increase of resource exploitation depths, multi-layer winding hoists have become the most suitable lifting equipment for ultra-deep coal mines in China. Wire rope, as a critical component in the multi-layer winding hoisting system, plays an important role in the process of mine safety production due to its unique mechanical properties (high axial strength and flexibility in bending). Therefore, the rope performance determines the hoisting capability and efficiency of the multi-layer winding hoist. However, in the process of ultra-deep coal mine hoisting, the hoist conditions are severe (hoist height is more than 1700 m, hoist speed is more than 15 m/s, and the hoist weight is more than 40 ton), and the existence of vibration in the hoisting system will cause reciprocating sliding, serious extrusion, and torsion of the wire rope. Thus, the friction and wear behavior between adjacent ropes always occur on the hoist drum, resulting in plastic deformation, crack, and fatigue fracture of the wires in the service rope. Furthermore, that damage to the rope will happen periodically as it winds on and off the drum continuously, which together determine its safety use and reduces the service life. Additionally, according to the coal mine safety rules in China [1], the number of winding layers of the wire rope on the drum must be one layer when lifting people in a vertical shaft and two layers when lifting materials alone, which is in contradiction with the necessity of multi-layer windings for an ultra-deep coal mine and seriously hinders the progress of its resource exploitation. The main reason is that with the number of winding layers increasing (more than two layers), the rope is easy to generate empty groove, rope skipping, interference, and other rope disorder phenomenon on the drum. Additionally, the friction characteristics and wear mechanisms caused by that damage behavior between the layers and adjacent ropes in the same layer have not been understood completely. Furthermore, the surface of a rope is very complicated due to its space helix layered structure, which makes the contact and wear become diversiform and complex. Therefore, it is of great significance to investigate the tribological characteristics of the hoisting rope, which can provide the basic data for the design of wire rope and multi-layer winding hoists in an ultra-deep coal mine.

In recent years, many studies have been carried out to investigate the performance of wire rope. Considering the complexity of the hierarchical helical structure, Wand et al. [2] analyzed the geometric construction of wire rope, and the mathematical model of ropes with different strand directions and types (right/left lang lay, wire structure as single-helixes, double-helixes, and super-coiled configurations) were created. To study the structure of ropes in actual working conditions, Ma et al. [3] presented two methods for building a 3-dimensional geometry of a wire rope bent over a sheave, and the two methods can be easily extended to other kinds of helical-strand wire rope. Costello [4] deeply analyzed the structural characteristics and mechanical properties of wire rope, and the theory is still in use. Additionally, as the experimental research on wire rope requires specific, large, and expensive testing devices, many scholars established finite element models to simulate its mechanical properties, which can predict the rope behavior, evaluate its structure condition, and detect damage as the rope is subjected to the working load [5,6,7]. Moreover, the failure and damage of wire rope are varied in different working conditions. Chaplin [8] discussed the degradation mechanisms of wire rope in service for three different applications, and the failure mechanisms of a mine hoist rope operating on a drum winder were analyzed. Mahmoud [9] investigated the fracture strength of a cracked bridge cable wire, and the surface crack and stress-strain curve were studied to forecast the wire degradation. Singh et al. [10] studied the causes of failure behavior of wire rope used in underground coal mines, and a physical examination, wear and corrosion, lubrication, macro and micro-examination, and chemical composition were selected for investigation. However, for multi-layer winding hoisting systems, the extrusion and wear between ropes are the major failure forms. To understand the tribological properties of the rope, Cruzado et al. [11,12] investigated the influence of crossing angle and contact pressure on the fretting wear of thin steel wires through a series of friction tests, and the evolution of the friction coefficient, wear scar surface, and wear volume were analyzed. Taking into account the degradation that occurs between the wires and the strands when the rope bends over a sheave, Urchegui et al. [13] designed the bending over sheave tests, obtained the wear evolution along the number of cycles, and the effect of the sheave diameter on the wear rate of a stranded rope employed in hoisting devices was analyzed. Furthermore, based on the experimental results, Argatov et al. [14] developed the mathematical models of fretting wear with application to the wear evolution between steel wires and applied Archard’s wear law, and the fatigue life estimations of ropes were analyzed. Wang et al. [15] studied the role of displacement amplitude on fretting fatigue behavior of the hoisting rope wires in low cycle fatigue, to simulate the wear damage under actual working conditions; three corrosive media were taken into account to quantitatively analyze the fretting fatigue damages of steel wires. Additionally, Xu et al. [16] carried out many fretting wear tests of steel wires in acid medium, and the fretting running characteristics, friction coefficient, dissipated energy, and wear morphology were analyzed. McColl et al. [17] examined the influence of low viscosity oils, with and without graphite additions, on the fretting behavior of the as-drawn wire. Oksanen et al. [18,19] studied the wear mechanisms at the wire rope groove surface of nodular cast iron rollers from rope drives, and the cracks caused during the wear processes were also analyzed. Chen et al. [20] established a numerical model for the interwire wear evolution of the strand subjected to a cyclic bending load based upon the thin rods theory, elastic contact theory, and Archard’s theory of wear, and the frictional contact and sliding between the wires were also considered. Moreover, considering the materials of rope wires, studies on the frictional characteristics between steel and steel are instructive for the research of wire rope. Hirsch et al. [21] investigated the influence of temperature on the fatigue damage in 301 stainless steel sheets due to fretting against 52100 steel and the changes in the material resistance as the temperature was increased from 20 to 250 °C were elucidated. Velkavrh et al. [22,23] analyzed the effects of different technical gases on the friction and wear behavior of steel contacts under severe operating conditions (non-lubricated sliding under high contact pressure, ambient and high temperature). Pearson et al. [24] investigated the effect of temperature (between 24 °C and 450 °C) on the wear rate and friction coefficient of a high strength alloy steel in gross sliding fretting in air. Mbarek et al. [25] developed an experimental study based on a twin-disc test configuration and the friction coefficient, temperature evolution, and wear in the regions close to the pitch point of the contact between the teeth of a polyamide-steel gear were investigated. However, most of the studies on friction are based on an experimental test which will require a high consumption of time and economic losses, thus, simulation research for the friction and wear behavior becomes very necessary. With the aim to reduce this disadvantage, Cruzado et al. [26,27] developed an optimized finite element wear simulation model for the simulation elliptical fretting wear scars in thin steel wires with different crossing angles, and the results were validated in comparison with experimental data obtained in laboratory fretting tests. Argatov et al. [28] employed an asymptotic modelling approach for solving the reciprocating sliding wear contact problem with an increasing contact zone under a prescribed constant normal load, and the obtained analytical results were compared with finite-element simulation results. Nevertheless, from the studies mentioned above, previous efforts mainly focus on the helical structure, the mechanical properties of wire rope, and the fretting wear behavior between rope wires. Additionally, there are only a few studies about the sliding friction and wear characteristics of ropes [29,30], and the effect of surface structure on the contact region, and the tribological properties of ropes in multi-layer winding hoists for ultra-deep coal mines have not previously been reported.

The aim of this paper is to investigate the effects of strand lay direction and crossing angle between ropes on the friction and wear behavior of wire rope used in multi-layer winding hoists. We consider that the displacement amplitude between ropes is usually larger than 300 μm, which belongs to reciprocating sliding [31,32]. Therefore, a series of sliding friction experiments on the wire rope samples’ cross contact along with different directions and angles have been carried out using a custom test rig. Furthermore, the parameters of the friction coefficient, friction temperature rise, wear mass loss, and wear scar morphology were analyzed. This study is helpful for the better design and rational use of the wire rope, which are very significant to improve its service life and performance. Additionally, the research results will provide basic data for the design of the multi-layer winding hoist in an ultra-deep coal mine.

## 2. Experimental Details

### 2.1. Materials and Specimens

**Figure 1:**
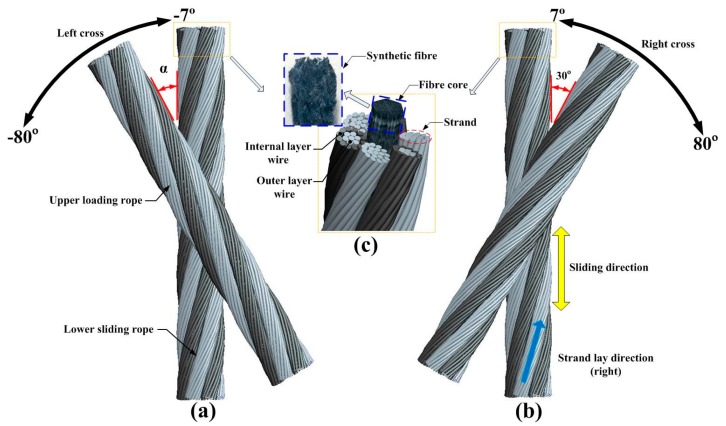
The 6 × 19 + FC wire rope specimens and the contact forms between them. (**a**) Left cross contact; (**b**) right cross contact; (**c**) structure details of the wire rope.

Figure 1 presents the structure details and the cross contact forms of wire rope tested in the experiments. The rope specimens are 6 × 19 + FC (Fibre core) (six strands with nineteen individual wires in each one, the core material is synthetic) point contact rope as shown in Figure 1c. Due to fibre cores having the advantage that they can store a relatively large amount of lubricant, the strands are supported softly, and this kind of rope should be well sounded and without any knots [33]. Thus, it is widely used in the winding hoist. Additionally, the material is the galvanized steel wires with a smooth surface that are manufactured by the coal drawing process from high quality carbon structural steel, and there is no lubrication between the contact ropes. Furthermore, as the lower sliding rope remains upright, the cross direction between two contact ropes can be divided into left cross as shown in Figure 1a (the cross direction and strand lay direction are opposite) and right cross as shown in Figure 1b (the cross direction is the same with the strand lay direction), taking into account the strand lay direction. The two contact forms will affect the tribological properties of ropes, which is what this paper focuses on. Moreover, other detail parameters of the test specimens are listed in Table 1.

### 2.2. Sliding Friction Test Rig and Test Conditions

**Figure 2:**
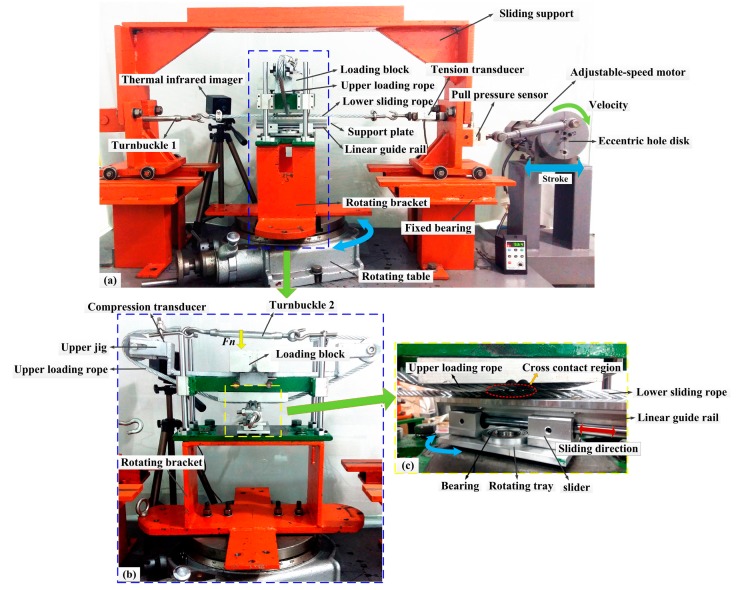
Sliding friction test rig. (**a**) Overall structure of the test rig; (**b**) structure of loading and rotation devices; (**c**) details for the contact condition between the rope specimens.

The structure of the self-made sliding friction test rig is illustrated in Figure 2, which evolved from the crank-slider mechanism. It can realize the sliding friction between the upper loading rope and the lower sliding rope under different displacement amplitudes, velocities, cross directions, and crossing angles. As shown in Figure 2a, the test rig mainly includes the driving device, sliding device, tensioning device, rotating device, and some transducers. Furthermore, each test needs two rope specimens, the lower sliding rope is strained on the sliding support that can achieve reciprocating motion by the driving of the adjustable-speed motor, and the upper loading rope is fixed in the upper jig which is match connected with the slide guide fixed on the rotating bracket, as shown is Figure 2b. Additionally, the liner guide rail and the slider are located under the lower sliding rope. The rotating tray is fixed together with the slider and can rotate freely through the bearing fixed on the rotating bracket. Therefore, the position of the lower sliding rope is almost constant when the rotating table rotates, and then the cross contact between the two ropes under different loads and crossing angles is achieved. Moreover, the tension of the rope specimens is adjusted through turnbuckles, tension transducers, and compression transducers. The friction force and friction temperature can be obtained using the pull pressure sensor and the thermal infrared imager, respectively. A detailed introduction of the test rig can be found elsewhere [30].

Considering the actual hoisting conditions in multi-layer winding hoists for ultra-deep coal mines, different experimental conditions were designed. In order to better understand the effects of strand lay direction and crossing angle on the tribological properties of wire rope, all the experiments can be divided into two major categories, left cross and right cross. Furthermore, the crossing angles of each category are in the range of 7–80°. Taking into account the characteristics of winding and disorder of ropes, the velocity and stroke will increase when the crossing angle become larger. Therefore, when the crossing angle is less than 30°, the velocity and stroke were selected as 6 mm/s and 10 mm, respectively. Otherwise, the velocity and stroke are 12 mm/s and 20 mm. Therefore, each category test can be divided into two groups, small crossing angle (from ±7° to ±28°) and large crossing angle (from ±40° to ±80°). Additionally, the severe wear always occurs under a poor lubricating condition, and dry friction for all the tests was selected. To reduce the test error caused by various factors, each experiment is repeated three times and the final result is the average value. The detailed parameters and conditions of each test are displayed in Table 2.

### 2.3. Test Parameters and Methods

**Figure 3:**
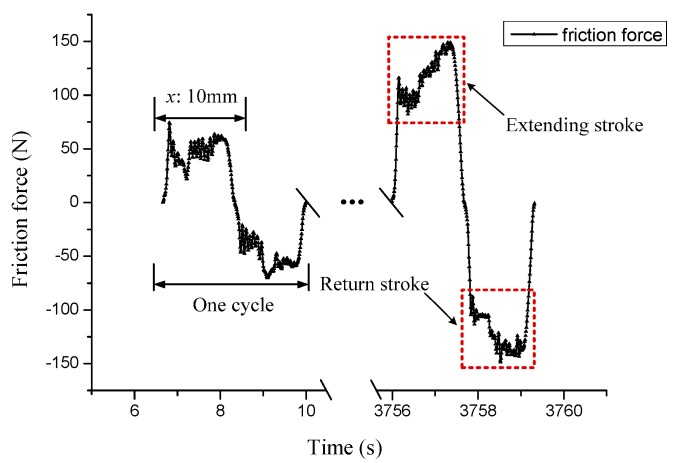
Friction force curve in the case of the right cross, and the crossing angle is 28°. The extending stroke and return stroke are marked by the dashed lines.

The friction coefficient, friction temperature rise, wear mass loss, wear scar, and its surface morphology are taken as the evaluation parameters to study the tribological properties of winding hoist rope under different cross directions and crossing angles. The evolution of the friction force can be recorded using a computer acquisition system during each experiment, as shown in Figure 3. It is clear that each cycle includes an extending stroke and return stroke because the sliding is a reciprocating motion. The data values in extending stroke are positive and negative in the return stroke. As the surface of the rope consists of steel wires and strands, the friction pair is irregularity which leads to the fluctuation of the curve in each stroke. Nevertheless, with the increase of test time, the curve become more and more stable and the value of the force become larger, thus, the change of the friction force curve is able to reflect the friction and contact state between the ropes. Furthermore, the mean friction coefficient *f_av_* in this paper is calculated only from the values in each extending stroke following the equation:

(1) $$f_{av}=\frac{\sum_{i=1}^{k}F_{fi}}{F_{n}k}$$

where *F_f_* is the friction force collected during each extending stroke, *F_n_* is the contact load, and *k* is the number of collected points in a single extending stroke. Therefore, the variation of the mean friction coefficient with the cycles was obtained.

**Figure 4:**
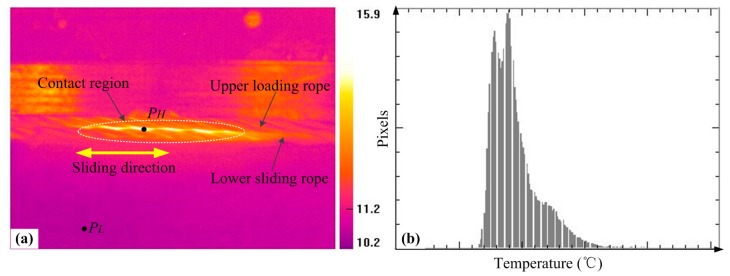
The infrared thermogram (**a**) and temperature distribution histogram (**b**) in the case of the right cross and the crossing angle is 7°.

The change of the friction temperature can affect the friction and wear response for materials, in particular for the wear debris between the contacting surfaces. To understand the friction behavior completely, the variation characteristics of the friction temperature rise were studied using a thermal infrared imager, which can complete the real-time monitoring, recording, and display for the temperature condition in the contact region. Figure 4 presents the pictures captured by the thermal infrared imager, which can intuitively reflect the change of friction temperature rise and the contact region through the distribution of color in the infrared thermogram. Furthermore, the evolution of the temperature value of the point selected in the picture can be derived through the software (ThermoX, MAGNITY ELECTRONICS, Shanghai, China), which is the counterpart of the thermal infrared imager. Therefore, the distribution of the temperature values can also be achieved. Moreover, the temperature rise Δ*T* studied in this paper is calculated by the highest temperature (*P_H_*) in the contact region subtracting the room temperature (*P_L_*).

Finally, the wear scar morphology characteristics were investigated by an industrial microscope. To evaluate the degree of wear quantitatively, all the debris of each test was collected and its mass was also measured using an electronic analytical balance with the measurement error less than 0.1 mg. The damage forms on the wear region and the sliding wear mechanisms were analyzed.

## 3. Results and Discussion

### 3.1. Evolution of the Friction Coefficient under Different Conditions

**Figure 5:**
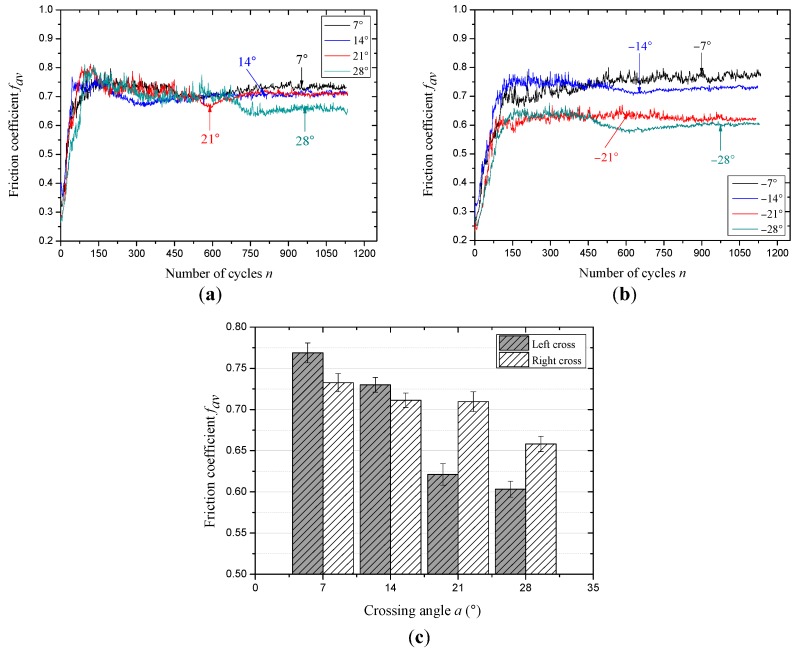
The evolution of the friction coefficient under different sliding conditions (*x*: 10 mm; *v*: 6 mm/s). (**a**) Right cross contact; (**b**) left cross contact and (**c**) the average values of the friction coefficient in the steady-state period.

The evolution of the friction coefficient between the wire rope under different cross directions and small crossing angles is presented in Figure 5. In general, the variation laws of the friction coefficient with cycles are similar, which can be divided into three stages (rapid growth stage, slow growth stage, and relatively stable stage), and it is a common change tendency for the friction coefficient. Figure 5a shows the change rules of the friction coefficient under different crossing angles with the contact condition that the cross direction is consistent with the strand lay direction (right cross). It is clear that as the number of cycles increases from 0 to 80, the friction coefficient increases rapidly to about 0.7; then, its growth speed slow down. When the cycles increase from 150 to 600, the friction coefficient presents a slight decreasing trend and the fluctuation of the curve is obvious. Finally, as the number of cycles continues to increase, the friction coefficient gradually stabilizes to different constants. Additionally, when the crossing angles are 14° and 21°, the corresponding curves are almost overlapping, indicating that the contact conditions are similar (the detailed explanation is provided in Section 3.3). Figure 5b presents the variation characteristics of the friction coefficient with the increasing sliding cycles under the condition of different crossing angles and left cross contact. As can be seen, the three stages and the difference between the curves are more obvious, in particular for the slow growth stage, and the process is more smooth although the fluctuation of the curves is still obvious. It indicates that the contact state is more stable and the wear evolution is more gentle. Additionally, the four curves completely separate and stabilize to different constants when the sliding is more than 750 cycles; this is due to the difference of the friction pair and a dynamic balance of wear between the ropes is finally achieved. Furthermore, the friction coefficient calculated from the values in the relative steady-state (about the last 300 cycles) under different crossing angles and cross directions is shown in Figure 5c. It can be observed that the friction coefficient in the relative steady-state showed a decreasing tendency with increasing crossing angles no matter what the cross direction is. However, when the cross direction between the ropes is left, the friction coefficient decreases from about 0.77–0.60 while the crossing angle increases from −7° to −28°; for the other condition, the average value decreases from approximately 0.73–0.66. Additionally, as the crossing angles are 14° and 21°, the values are almost equal, approximately 0.71.

**Figure 6:**
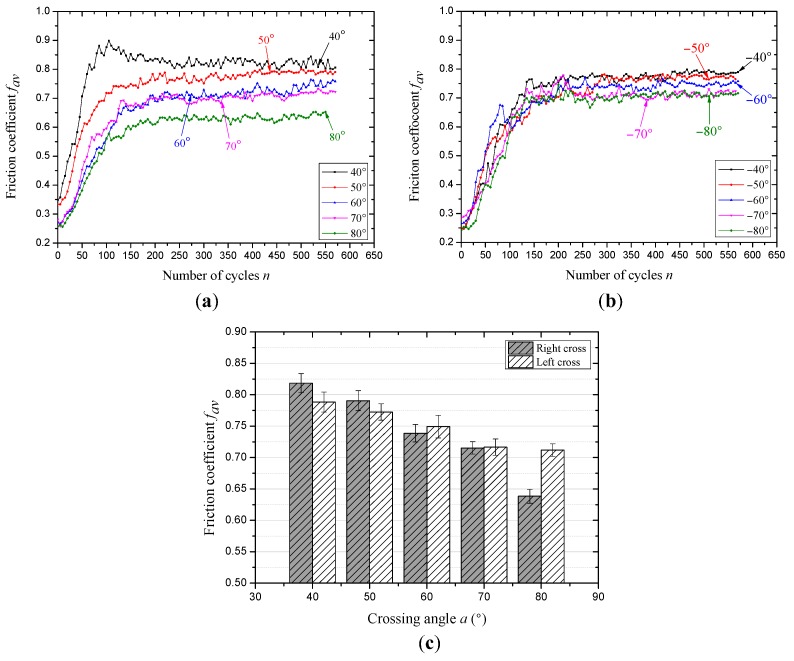
The evolution of friction coefficient under different sliding conditions (*x*: 20 mm; *v*: 12 mm/s). (**a**) Right cross contact; (**b**) left cross contact and (**c**) the average values of the friction coefficient in the steady-state period.

Figure 6 presents the variation of the friction coefficient under the condition of different cross directions and large crossing angles. Because to the velocity and stroke are increased to 12 mm/s and 20 mm, the number of sliding cycles is half of that in Figure 5. Under the contact condition of right across, the effect of the large crossing angle on the friction coefficient can be observed in Figure 6a. It is clear that the difference between each curve is obvious and there are almost no overlapping curves, which indicates that the crossing angle plays an important role in this condition. The rate of the curve increases with the increasing crossing angles during the early stage (approximately 100 cycles), then, after a slow transition stage for about 125 cycles the curves finally stabilize to different constants. The other case is the effect of the large crossing angle under the condition of left cross, as can be seen in Figure 6b. Compared with Figure 6a, the evolution of the friction coefficient is more similar and the overlapping of different curves is more obvious. This indicates that the surface between the friction pair is similar and the effect of the crossing angles on the contact surface is small under left cross. Moreover, the number of cycles to each friction stage is probably identical, indicating that the process of wear is mainly affected by the sliding distance. Furthermore, the friction coefficient in the relative steady-stage (after about 450 cycles) under the condition of different crossing angles and cross directions is shown in Figure 6c. For the two cross directions, the friction coefficient in the stable stage shows a decreasing trend with the increasing crossing angles. It decreases from about 0.82–0.64 and 0.79–0.71 under the contact condition of right cross and left cross, respectively. Therefore, the crossing angles have a more obvious effect on the friction coefficient under the condition of right cross.

The evolution process of the friction coefficient can reflect the change of friction surfaces. Because the rope wires are galvanized and there is no lubrication between the contact surfaces, the friction pair is smooth and the damage is fast at the beginning of each test, which lead to rapid growth in the early stage. When the crossing angle is small (±7° to ±28°), the contact surface is similar under the condition of right cross, because the cross direction and the strand lay direction are the same, and the sliding will occur in the gap between the rope strands, which can be found in the Section 3.3. For Figure 6, as the stroke is 20 mm and the strand lay length is 70 mm, the reciprocating sliding will occur between the two gaps, which lead to a larger friction force. Additionally, with the increase of the crossing angle, the difference between the contact surfaces is great and can be observed in Section 3.3. Compared with the other case (left cross contact), the effect of the surface structure on the friction coefficient is not obvious, and the contact state is relatively more stable.

### 3.2. Evolution of Temperature Rise under Different Conditions

**Figure 7:**
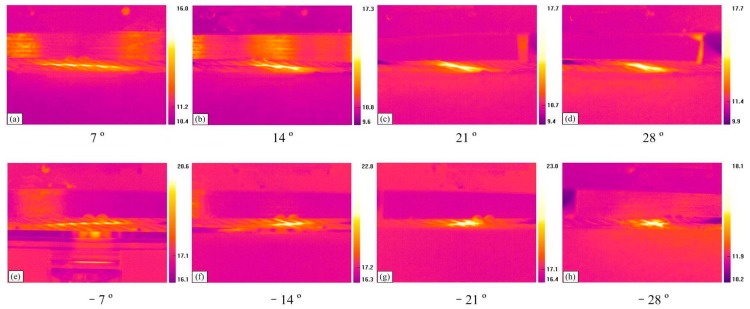
Infrared thermograms for different sliding conditions (*x*: 10 mm; *v*: 6 mm/s). (**a**–**d**): Right cross contact; (**e**–**h**): left cross contact.

Figure 7 shows the infrared thermograms captured before the end of the friction tests under the sliding conditions of small crossing angles and different cross directions. It is clear that the color in the contact region is brighter, indicating that the temperature rise caused by the friction between ropes is very obvious; the brighter the color, the higher the temperature. A general trend can be seen in that the temperature rise in the sliding region increases with the crossing angles. Furthermore, those images can intuitively reflect the variation and characteristics of the contact areas. The infrared thermograms for the conditions of different crossing angles and right cross can be observed in Figure 7a–d. When the crossing angle increases from 7° to 28°, the contact areas become increasingly concentrated and the high temperature regions gradually distribute along the strand lay direction. However, compared with the other contact condition, as can be seen in Figure 7e–h, it is obvious that the high temperature regions are continuously concentrated with the crossing angle, and distribute in the middle of the contact area. It indicates that the effect of cross direction between the rope samples on the high temperature regions’ distribution and contact surface is very obvious.

**Figure 8:**
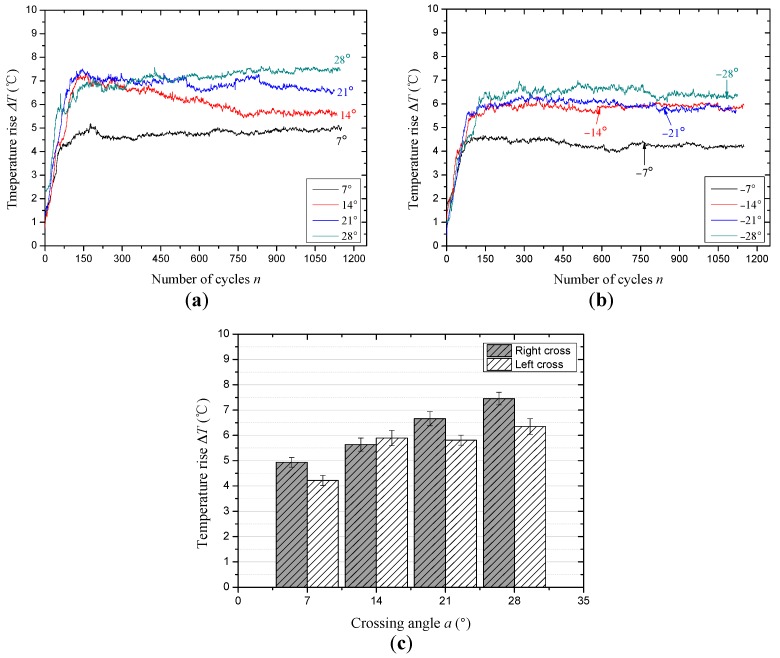
The evolution of the temperature rise under different sliding conditions (*x*: 10 mm; *v*: 6 mm/s). (**a**) Right cross contact; (**b**) left cross contact; (**c**) the average values of the temperature rise in the steady-state period.

Figure 8 presents the evolution of friction temperature rise between the wire ropes under the sliding conditions of different cross directions and small crossing angles. Similar to the change law of the friction coefficient, the temperature rise does not increase during the whole friction test, but increases rapidly for a short time at first, and then gradually tends to be stable. Additionally, the distinction between each temperature rise curve is clearer. As the cross direction is right, the effect of the crossing angle on the temperature rise can be obtained from Figure 8a. At the beginning of each test (before approximately 150 cycles), the temperature rise increases rapidly and reaches a maximum value. In the next 150 sliding cycles, the overlapping phenomenon of each curve is serious, indicating that the severe wear stage will end. Finally, the curves reach a stable stage after about 800 sliding cycles, which illustrates that the friction temperature rise has realized a relative balance state between the generation and dissipation of the heat. Figure 8b presents the evolution of the temperature rise under the condition of left cross for small crossing angles. It is clear that the temperature rise curves reach the relatively stable stage earlier, approximately 500 sliding cycles. It indicates that the sliding surface and the process of wear are more stable. Moreover, Figure 8c presents the variation of the average temperature rise calculated from the values in the stable stage (about the last 350 cycles) under the condition of different cross directions and crossing angles. When the cross direction is the same as the strand lay direction, the temperature rise in the steady-state period is higher than that for left cross. With the crossing angle increases from 7° to 28°, the temperature rise increases from approximately 4.9 °C to approximately 7.5 °C and from approximately 4.2 °C to approximately 6.3 °C under the condition of right cross and left cross, respectively.

**Figure 9:**
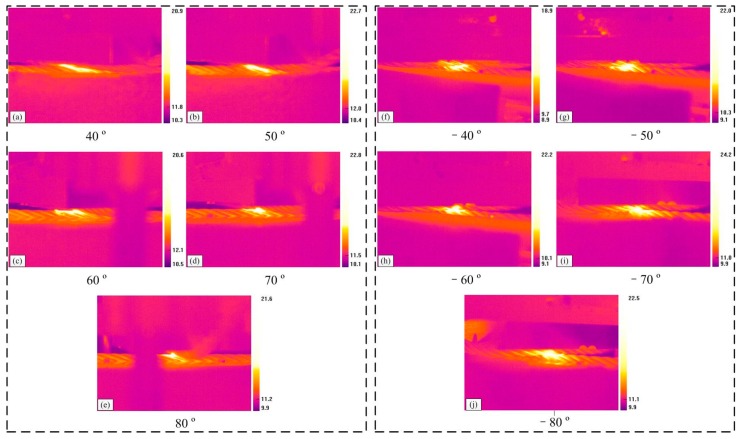
Infrared thermograms for different sliding conditions (*x*: 20 mm; *v*: 12 mm/s). (**a**–**e**): Right cross contact; (**f**–**j**): left cross contact.

Figure 9 presents the infrared thermograms captured before the end of the friction tests under the sliding condition of large crossing angles and different cross directions. Compared with Figure 7, the variation characteristics of the temperature rise distribution in the sliding regions for right cross are different, as can be seen in Figure 9a–e. When the crossing angle is 40° and 50°, the high temperature areas distribute among the gaps between strands along with the strand lay direction. However, as it increases from 60° to 80°, the high temperature areas become more concentrated and distribute on the upper surface of the strands, indicating that the effect of the strand lay direction on the contact region becomes smaller when the crossing angle is larger. However, for the other case, the change of the high temperature area is not very obvious, as shown in Figure 9f–j. Additionally, when the crossing angle increases from −40° to −80°, the color in the contact region becomes brighter. Compared with the two cross conditions under large crossing angles, the effect of the strand lay direction on the high temperature area distribution is more obvious for the right cross contact, but for the left cross contact, the crossing angle plays an important role in the temperature rise.

**Figure 10:**
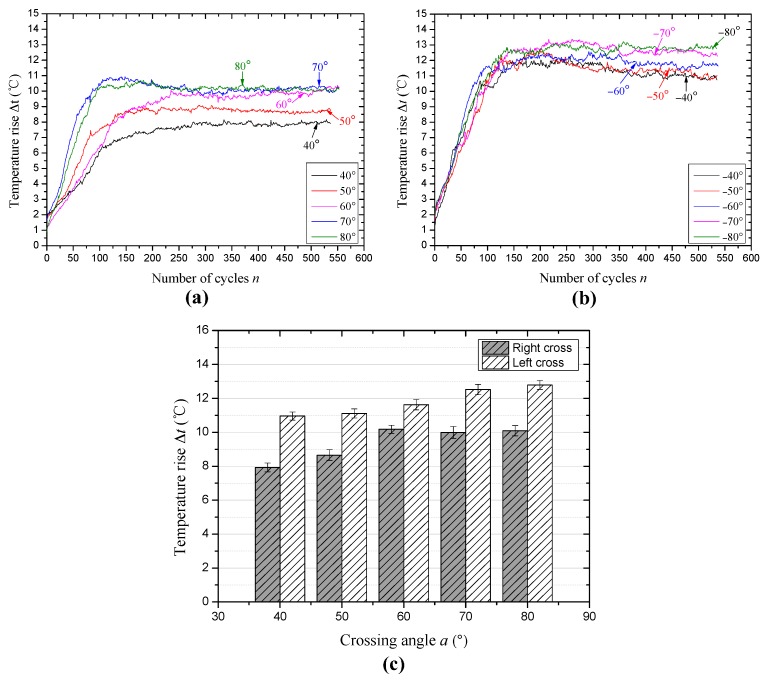
The evolution of the temperature rise under different sliding conditions (*x*: 20 mm; *v*: 12 mm/s). (**a**) Right cross contact; (**b**) left cross contact and (**c**) the average values of the temperature rise in the steady-state period.

The evolution of friction temperature rise between wire rope under the condition of different cross directions and large crossing angles is presented in Figure 10. The variation trend of the curves is very similar to Figure 6, when the cross direction is the same with the strand lay direction as shown in Figure 10a, the difference between each temperature rise curve is more obvious, in particular for the early stage. This illustrates that contact properties are different at first under different crossing angles. Additionally, when the crossing angle is 70° and 80°, this two temperature rise curves are very similar, and as the temperature rise achieves relative stable, there are three curves overlap with each other which crossing angle is 60°, 70° and 80°, respectively. It is correspond to the result shown in Figure 9c–e. Figure 10b presents the variation of temperature rise under the condition of left cross contact and large crossing angles. Different from Figure 10a, the curves with different crossing angles are disorder at the beginning of the tests, which are difficult to distinguish each curve. But after the temperature rise becomes stable, the curves with different crossing angles are easy to distinguish. It indicates that the wear processes in the early stage (about 150 sliding cycles) are similar and gradually stabilize to different balance stages under the effect of different crossing angles. The average values of temperature rise during the relative steady-state (approximately the last 150 cycles) with different crossing angles and cross directions are shown in Figure 10c. It is clear that with the increase of crossing angles, the temperature rise in the contact region increases from approximately 7.9 °C to approximately 10.1 °C and approximately 10.9 °C to approximately 12.8 °C under the condition of right cross and left cross, respectively. Additionally, as the cross direction is opposite to the strand lay direction, the temperature rise is higher than that for right cross under the same crossing angle.

In this study, the temperature rise is caused by the friction heat between wire ropes, which will influence the characteristics of the sliding contact surface, especially for the wear debris between the friction surfaces. However, the temperature rise in the contact region is not so obvious in the actual working condition, but, the transient temperature that arises is large and the temperature of asperities may be much higher than that of the surface, which then forms local high-temperature zones [34]. Additionally, the infrared thermograms can intuitively reflect the variation of contact types between rope samples and through the distribution of the color, which in turn presents the effect of cross direction under different crossing angles on the friction heat and sliding contact. Furthermore, the evolution of the temperature rise is the changing process of surface wear. In the early stage, the damage of the friction surface is very fast and the contact region is extending, which will generate a lot of friction heat. However, as the wear becomes stable, the heat generation and dissipation realize a dynamic balance state gradually. Additionally, it shows a contrary tendency with the variation of the friction coefficient, which indicates that the temperature rise in the relatively stable state increases with the decrease of the contact area. With the increase of the crossing angle, the contact pressure becomes larger, which is more beneficial to increase the friction temperature rise. This result corresponds to the previous research [31]. However, due to the difference of the cross directions, the average temperature rise in the relative steady-state is different despite the variation tendency with increasing crossing angle being similar.

### 3.3. Wear Mechanism

**Figure 11:**
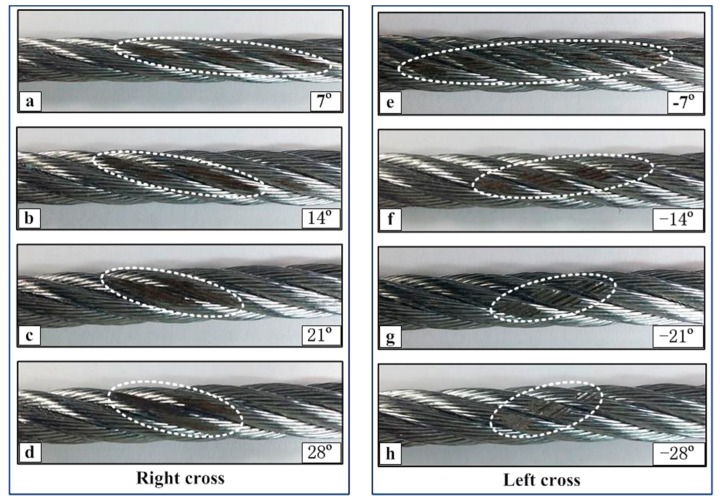
Wear scar pictures of the upper loading rope for a small crossing angle under different sliding conditions (*x*: 10 mm; *v*: 6 mm/s). (**a**–**d**): Right cross contact; (**e**–**h**): left cross contact. The wear scar regions are marked by the dashed lines.

**Figure 12:**
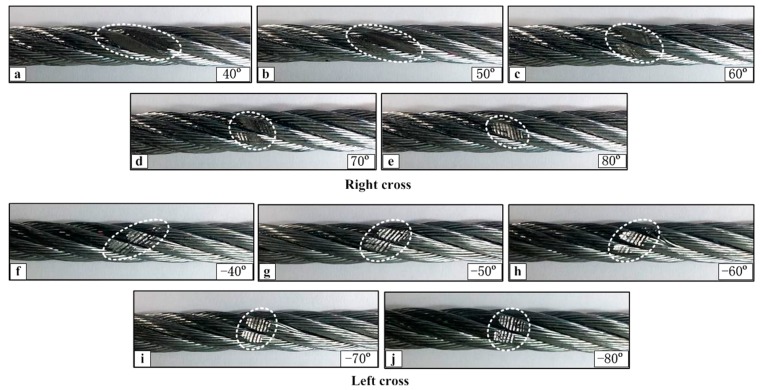
Wear scar pictures of the upper loading rope for a large crossing angle under different sliding conditions (*x*: 20 mm; *v*: 12 mm/s). (**a**–**e**): Right cross contact; (**f**–**j**): left cross contact. The wear scar regions are marked by the dashed lines.

**Figure 13:**
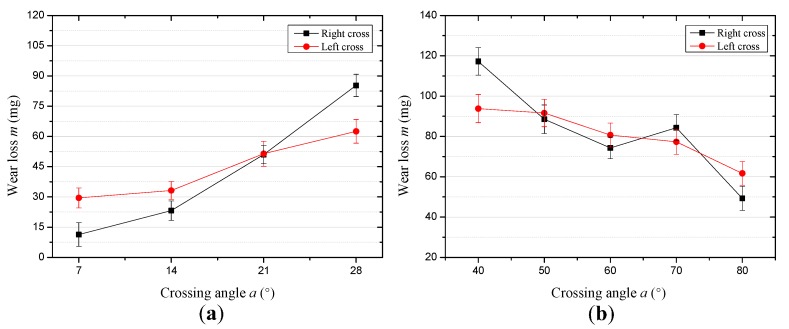
Wear mass loss for the different sliding conditions. (**a**) The wear mass loss for different cross directions in the case of a small crossing angle (*x*: 10 mm; *v*: 6 mm/s) and (**b**) the wear mass loss for different cross directions in the case of a large crossing angle (*x*: 20 mm; *v*: 12 mm/s).

**Figure 14:**
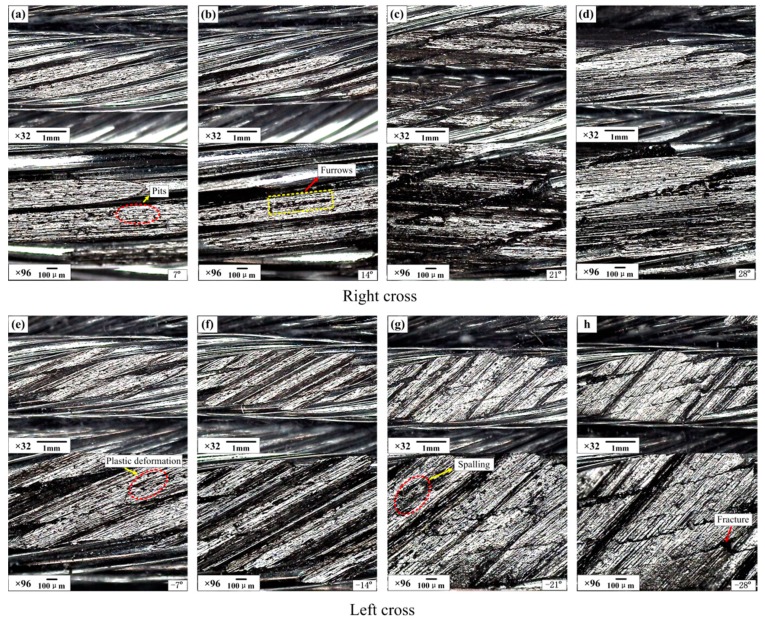
Industrial microscope images of the wear surface of the upper loading rope for different conditions (*x*: 10 mm; *v*: 6 mm/s). (**a**–**d**): Right cross contact; (**e**–**h**): left cross contact.

**Figure 15:**
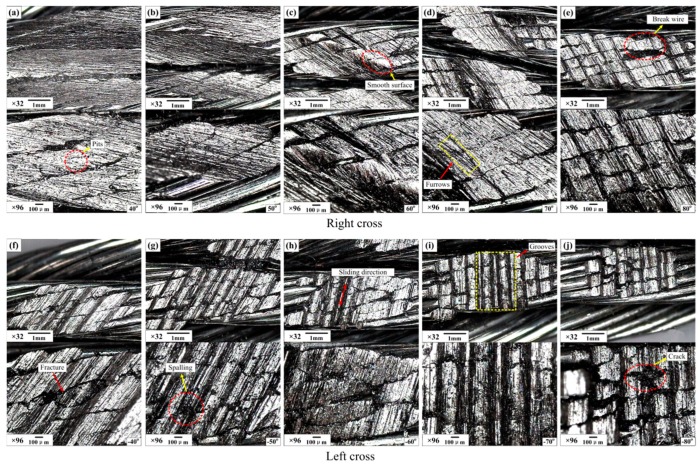
Industrial microscope images of the wear surface of the upper loading rope for different conditions (*x*: 20 mm; *v*: 12 mm/s). (**a**–**e**): right cross contact; (**f**–**j**): left cross contact.

The effect of the strand lay direction and crossing angle on the sliding contact characteristics were investigated by a visualization method, and the wear scars are provided in Figure 11 and Figure 12. Additionally, the wear mass loss for each test is presented in Figure 13 to quantitatively analyze the degree of wear. Furthermore, the worn surface micrographs of the upper loading ropes, as can be seen in Figure 14 and Figure 15, are studied to understand the wear mechanisms and provide explanations for the variation laws of the friction coefficient and temperature rise.

Figure 11 presents the wear scar pictures of the upper loading ropes under different cross directions and small crossing angles. In the case of right cross, the wear scar region becomes more concentrated with the crossing angle. When the crossing angle is 7°, the wear scar occurs on many strands and its width is small. However, when the crossing angle is more than 14°, the wear scar only occurs on two strands and distributes in the gap between the side surfaces of the strands. Compared with the other case, as shown in Figure 11e–h, the differences are obvious. It is clear that the wear scars always appear on many strands with the increase of the crossing angles. At the crossing angle of 7°, the distribution of the wear scar is similar to that in Figure 11a, but, with the increase of angles, the wear scars become more and more obvious and only appear on the upper surface of the strands. This corresponds to the distribution of high temperature areas as shown in Figure 7.

The wear scar pictures of the upper loading rope under the condition of different cross directions and large crossing angles are shown in Figure 12. In the case of right cross, the change of the wear scar profiles are larger than that in the case of left cross with an increase of the crossing angle. When the crossing angle is 40° and 50°, the wear scar still distributes on the side of the adjacent strands, which is the reason why the high temperature distributes along the strand lay direction shown in Figure 9a,b. However, when the angle is more than 60°, the wear area changes from the gap between the adjacent strands to the upper surface of the strands. Additionally, its shape becomes more regular and small. However, for the case of left cross, the shape of the wear area is very clear and surface characteristics are similar under different crossing angles. Comparing with Figure 11a–e, the contact form is singular and stable, and the effect of the crossing angle is more obvious. This is the reason why the variation of the friction coefficient and temperature rise in the steady-state is small for this contact condition.

Figure 13 presents the evolution of the wear mass loss for each test. It can be concluded that in the case of the small crossing angle with the stroke and velocity at 10 mm and 6 mm/s, respectively, the wear loss increases from about 11.3–85.3 mg and 29.5–62.5 mg under the condition of the right cross contact and left cross contact, as can be seen in Figure 13a. However, as the crossing angle increases from 40° to 80°, the wear loss decreases from approximately 117.2–49.3 mg under the condition of right cross and decreases from about 93.8–61.7 mg in the other condition. It indicates that in the condition of right cross between the wire ropes, the variation of the wear mass loss is larger than that for left cross with the crossing angle, even though the changing trend is similar. Therefore, the strand lay direction plays an important role in the wear mass loss.

Figure 14 shows the worn surface micrographs of the upper loading rope achieved using an industrial microscope under different cross directions and crossing angles. It is obvious that there are many similar morphological features of the wear surface for all the tests, such as the pits, furrows, plastic deformation, and spalling fatigue that can be found easily on the images of partially enlarged details. From Figure 14a–d, it can be seen that when the crossing angle is 7° and 14°, there is a mild wear on the upper surface of the strands and the gaps between the rope wires are very obvious. Additionally, the wear characteristics are mainly pits and plastic deformation, because the contact regions are larger and the contact pressure is relatively smaller. However, when the crossing angle increases to 21° and 28°, the wear becomes more acute and the surface is more complete. As shown in Figure 14d, both sides of the strand surface are damaged and the wear scar surface is not in the same plane due to the sliding extrusion of the strands in the lower sliding rope. Furthermore, the furrows are more intensive and complete as shown in Figure 14d. This is because the contact pressure is larger which leads to a closer contact. Considering the effect of wear debris, it is easier to stay on the contact surface as the wear scar distributes in the gap between the adjacent strands, which leads to the gaps between adjacent rope wires being smaller. Figure 14e–h present the images of the wear scar surface under the condition of left cross and different crossing angles. It is clear that the surface morphology is more complex than that for the right cross contact. As the crossing angle is −14°, there are many grooves caused by the external wires in the wear region, additionally, the plastic deformation and ploughing are serious which make the surface very rough. However, when the crossing angle increases to −28°, the honing pattern becomes more clear and complete. This is the reason why the friction coefficient shows a decreasing trend, as shown in Figure 5. Therefore, the major wear mechanisms are adhesive wear and abrasive wear in this contact condition.

Figure 15 shows the worn surface micrographs of the upper loading rope under the condition of the large crossing angle and different cross directions. Comparing with Figure 14, the degree of wear is more severe and the variation of the wear morphology is more obvious. When the cross direction is the same as the strand lay direction, as can be seen in Figure 15a–e, the wear scars can be divided into three types. As the crossing angle is 40° and 50°, the effect of the strand lay direction on the characteristics of the sliding contact is observable. It is clear that the severe wear always occurs on the side surfaces and there is a pointy embossment on the middle of the rope strand which is caused by the adjacent strands in the lower sliding rope. As the crossing angle increases to 60° and 70°, the damage on the wear surface is very serious and the furrows are disordered. Additionally, the wear scar regions move to the upper surface gradually and there is a relatively smooth surface on the larger embossment in the middle of the wear scar, as shown in Figure 15c,d. Finally, as the crossing angle increases to 80°, the wear region moves to the upper surface of the strand completely. Many grooves also occur in the region with a small amount of crack and spalling fatigue. Therefore, with the increase of the crossing angle, the wear region becomes smaller and the contact surface becomes more smooth, which provides a good explanation for the evolution of the friction coefficient, as can be seen in Figure 6. However, when the cross direction changes into left as shown in Figure 15f–j, all the wear scars are distributed on the upper surfaces of the strands and are almost not affected by the strand lay direction. It is obvious that the grooves are clear in the wear region for each test, indicating that the sliding tract is relatively more stable. Due to the increase of the crossing angle, the contact region becomes small and the wear depth becomes larger, and the phenomenon of fatigue fracture of the wires occurs on the wear surface as shown in Figure 15j. Additionally, the greater contact pressure is easier to cause closer contact and serious wear, which is helpful for the generation of friction heat. This is consistent with the variation rules shown in Figure 10. Therefore, the wear mechanisms are adhesive wear, abrasive wear, and fatigue wear in this sliding contact condition.

## 4. Conclusions

The present research on the tribological properties between winding hoisting ropes under different cross directions and crossing angles revealed the following findings:

- The friction coefficient in the steady-state period shows a decreasing tendency with an increase of the crossing angle in both cross directions. In the case of a small crossing angle (from 7° to 28°), the friction coefficient changes in a wider range under the contact condition of left cross, from approximately 0.77 to approximately 0.60. However, when the crossing angle is large (from 40° to 80°), the change of the friction coefficient is more obvious under the condition of right cross, from approximately 0.82 to approximately 0.64.
- The temperature rise in the steady-state period shows an increasing tendency with the crossing angles in both cross directions. In the case of right cross contact, the high temperature regions always distribute along the strand lay direction between the adjacent strands in most situations. Nevertheless, when the cross direction is left, the high temperature regions distribute on the upper surface of the rope strands. Additionally, when the crossing angle is small, the temperature rise is larger under the contact condition of right cross, increasing from approximately 4.9 °C to approximately 7.5 °C. However, in the case of a large crossing angle, the temperature rise is larger under the contact condition of left cross, increasing from approximately 7.5 °C to approximately 12.8 °C.
- The variation of wear scar with an increase in the crossing angle is different for different cross directions. In the case of right cross, the wear scar area gradually concentrates to the gap between the adjacent strands with the crossing angle increasing from 7° to 28°. Additionally, as the crossing angle increases from 40° to 80°, the wear scar area moves from the side surfaces in the gap to the upper surfaces of the strands. However, as the cross direction is left, the wear scar is always on the upper surfaces and becomes more concentrated with the increase of the crossing angle.
- The wear mass loss shows an increasing tendency in the small crossing angle tests and shows a decreasing tendency in the large crossing angle tests for both cross directions. The variation range of the wear mass loss is larger in the case of right cross contact.
- The differences of the wear morphology in the case of different cross directions are very obvious, in particular for the large crossing angle tests. The damage formed on the wear surface are mainly ploughing, pits, plastic deformation, and fatigue fracture. The major wear mechanisms between the winding hoist ropes in this study are adhesive wear, abrasive, and fatigue wear.

## Figures and Tables

**Table 1:** Parameters of the wire rope specimens.

Parameter	Value
Diameter of the rope	9.3 mm
Radius of the steel wires	0.3 mm
Strand lay length	70 mm
Strand lay angle	15.5°
Strand lay direction	Right
Nominal tensile strength	1570 MPa
Breaking force	52,500 N

**Table 2:** Sliding friction test conditions for the cross contact ropes.

Test Parameters	Left Cross		Right Cross	
Contact load (*F_n_*)	150 N	150 N	150 N	150 N
Crossing angle (*α*)	−7°; −14°; −21°; −28°	−40°; −50°; −60°; −70°; −80°	7°; 14°; 21°; 28°	40°; 50°; 60°; 70°; 80°
Stroke (*x*)	10 mm	20 mm	10 mm	20 mm
Number of cycles (*n*)	1136	568	1136	568
Velocity (*v*)	6 mm/s	12 mm/s	6 mm/s	12 mm/s
Tensile force (*F*)	2000 N	2000 N	2000 N	2000 N
Sliding distance (*s*)	22,720 mm	22,720 mm	22,720 mm	22,720 mm
Lubricant condition	Dry-friction	Dry-friction	Dry-friction	Dry-friction
Temperature	Room temperature	Room temperature	Room temperature	Room temperature
Humidity (%)	60 ± 5	60 ± 5	60 ± 5	60 ± 5
Atmosphere	Laboratory air	Laboratory air	Laboratory air	Laboratory air
